# High Velocity Passive Stretching Mimics Eccentric Exercise in Cerebral Palsy and May Be Used to Increase Spastic Muscle Fascicle Length

**DOI:** 10.3390/bioengineering11060608

**Published:** 2024-06-13

**Authors:** Jessica F. Davis, Tahir Khan, Matt Thornton, Neil D. Reeves, Mara DeLuca, Amir A. Mohagheghi

**Affiliations:** 1 Centre of Health, Physical Activity, Exercise and Rehabilitation, Brunel University London, Uxbridge UB8 3PH, UK; jessica.davis@brunel.ac.uk (J.F.D.); mara.deluca@brunel.ac.uk (M.D.); 2 The Royal National Orthopaedic Hospital, Stanmore HA7 4LP, UKmatt.thornton@nhs.net (M.T.); 3 Department of Life Sciences, Faculty of Science & Engineering, Manchester Metropolitan University, Stanmore HA7 4LP, UK; n.reeves@mmu.ac.uk; 4 Centre for Cognitive and Clinical Neuroscience, Brunel University London, London UB8 3PH, UK

**Keywords:** cerebral palsy, eccentric, fascicle length

## Abstract

Muscle fascicles are shorter and stiffer than normal in spastic Cerebral Palsy (CP). Increasing fascicle length (FL) has been attempted in CP, the outcomes of which have been unsatisfactory. In healthy muscles, FL can be increased using eccentric exercise at high velocities (ECC). Three conditions are possibly met during such ECC: muscle micro-damage, positive fascicle strain, and momentary muscle deactivation during lengthening. Participants with and without CP underwent a single bout of passive stretching at (appropriately) high velocities using isokinetic dynamometry, during which we examined muscle and fascicle behaviour. Vastus lateralis (VL) FL change was measured using ultrasonography and showed positive fascicle strain. Measures of muscle creatine kinase were used to establish whether micro-damage occurred in response to stretching, but the results did not confirm damage in either group. Vastus medialis (VM) and biceps femoris muscle activity were measured using electromyography in those with CP. Results supported momentary spastic muscle deactivation during lengthening: all participants experienced at least one epoch (60 ms) of increased activation followed by activation inhibition/deactivation of the VM during knee flexion. We argue that high-velocity passive stretching in CP provides a movement context which mimics ECC and could be used to increase spastic FL with training.

## 1. Introduction

Muscle fascicle length has been suggested as the single most important architectural parameter of a muscle affecting its function [1]. Many motor disabilities with neurological origin such as cerebral palsy (CP), which present clinically with spasticity and contracture, are associated with relatively shorter muscle fascicles compared to matched healthy controls [2,3,4,5] that could contribute to impaired movements often observed within this population. Assuming similar muscle lengths and tendon moment arms, shorter fascicles will mean that the sarcomeres experience a greater length change for the same range of motion, thereby forcing the sarcomeres to operate over less advantageous portions of their length–tension relationship [6]. Therefore, an increase in muscle fascicle length (FL) may be a desirable outcome for individuals with CP, as it could improve functional abilities by altering the length–tension and force–velocity characteristics of the muscle [7,8]. Increases in FL can also promote higher maximum shortening velocity and muscle power, shifting the optimum fibre length for contraction to greater joint angles due to an increase in serial sarcomere number (SSN) [9]. Moreover, an increase in the threshold for the tonic stretch reflex could be an additional outcome of increased FL due to the increase in the number of sarcomeres.

Despite several reports of increases in the length of muscle fascicles in response to various conservative interventions in healthy humans and animals [10,11], necessary and sufficient exercise conditions to consistently increase FL through increases in SSN in humans, and particularly in individuals with CP, are yet to be fully determined. Evidence from the literature supports the notion that long-term/chronic training may result in an increase in muscle FL in healthy animals and humans [10,12,13,14,15,16,17]; however, this is likely to occur when specific exercise and training conditions are provided [18]. For example, not all resistance training has been found to increase FL: Sharifnezhad et al. (2014) used four different eccentric protocols to establish training conditions necessary to increase FL in typically developed individuals (TD). In their study, the velocity of training and MTU length were manipulated whilst the training load was controlled. From the four protocols employed where the load was controlled, only eccentric training at the highest lengthening velocity (240 deg/s) resulted in increases in FL [10]. However, Marzilger et al. (2019) investigated the effects of lengthening velocity on the physiological cross-sectional area and FL and reported that all angular velocities resulted in similar increases in FL [15]. 

We previously proposed three criteria for an optimal training condition which may lead to the increase in FL in healthy muscles: (1) eccentric exercise at appropriately high velocity, leading to (2) microscopic damage of the stretched fascicles, and (3) associated with momentary deactivation of the elongated/stretched muscle during exercise [19]. Three measurable outcomes to examine the viability of our proposition, when exercising or training under these presumed optimal conditions include positive fascicle strain, increased level of a blood biomarker (e.g., muscle-CK) representing microscopic damage to muscle cells, and drop in muscle electrical activity (EMG) representing a period of reduced activity. Whether such (theoretical) optimal training conditions could be consistently induced in healthy muscles, measured using the outcome parameters above, and lead to an increase in the length of muscle fascicles has not been determined yet. The behaviour of spastic muscles and fascicles during exercise is even less understood [20,21,22,23], and it is not clear if the proposed optimal training conditions for healthy muscles can be successfully created in exercise laboratories to train spastic muscles and increase FL.

In individuals with CP, eccentric training of spastic muscles at high velocity may be limited by the lack of selective motor control [24]. Spastic muscles also show a characteristic response to stretching: an exaggerated velocity-dependent response, where larger reflex muscle activity is triggered at shorter lengths of the muscle [25,26]. Moreover, reflex contraction in spastic muscles may suddenly give way (clasp-knife phenomenon) if stretching continues [22]. Such a response to high-velocity stretching, if occurring in spastic muscles, however, would mimic eccentric training in healthy individuals under the presumed optimal condition proposed above, and may lead to an increase in FL. This could be extremely beneficial to those with CP as it could alter the length–tension characteristics of the muscle, meaning it will contract at a more optimal length. 

The present study was designed to provide preliminary information for assessing the viability of the suggested optimal training conditions above for increasing muscle FL in healthy muscles via providing insight into the behaviour of spastic muscle fascicles in response to high-velocity passive stretching, an exercise mimicking eccentric contraction in healthy muscles [19]. 

In the present study, we aimed to gain an insight into the spastic muscle and fibre behaviour in response to high-velocity passive stretching and assess if presumed conditions for increasing FL in healthy muscles discussed above could be met in a single experimental session in individuals with spastic CP. Due to the exploratory nature of the study, no formal hypotheses were formed. Since no previous information on the behaviour of spastic fibres under such experimental conditions was available, we used the minimal detectable change in CK as a measure of muscle microdamage in response to stretching in healthy muscles as a reference for spastic individuals. Moreover, we monitored fibre strain and established whether deactivation of the spastic muscle could happen during stretching. Experimenter reliability in the examination of FL was assessed in the healthy muscles to reduce the burden on the individuals with CP. 

## 2. Methods

### 2.1. Participants

A sample of convenience of 7 individuals with cerebral palsy (1F/6M; mean (SD) age = 21 (9) years) and 28 individuals without CP aged 18–50 years (TD) (14F/14M; mean (SD) mass = 76.63 (26) Kg; mean (SD) height = 167.5 (23.5) cm) volunteered to participate in this study. CP individuals were either hemiplegic (*n* = 2) or diplegic (*n* = 5), all level I-III on the Gross Motor Functioning Classification System, and were recruited through the Royal National Orthopaedic Hospital (RNOH). TD individuals were recruited from the students and staff of Brunel University London and were all free from lower limb neuromuscular injuries, blood-borne viruses, and metabolic conditions. Ethical approval for conducting this study was obtained from Brunel University London College of Health, Medicine and Life Sciences Research Ethics Committee (for study involving TD participants), and from the NHS Research Ethics Committee (for study involving individuals with CP). All participants provided written informed consent to participate in this study. 

### 2.2. Experimental Design

The CP group (Protocol 1) was recruited to monitor the behaviour of spastic vastus lateralis (VL) muscle fascicles during stretching at different velocities to examine whether the 3 presumed criteria for increase in the length of FL with exercise could be observed in an exploratory (non-confirmatory) one-off study. Similarly, the TD group were recruited to determine the immediate effect of stretching and reliability of measurements of the VL muscle FL and muscle-CK in a group of individuals without CP. 

CP participants attended the RNOH for gait analysis on an instrumented treadmill. Participants’ highest knee joint velocities during flexion were used for individualising experimental protocol. All participants then attended the Biomechanics Laboratory at Brunel University London on three consecutive days at the same time of day. TD participants were randomly allocated into either an exercise group (TD-EG) (Protocol 2) or a control group (TD-CG) (Protocol 3) according to their order of recruitment. Participants were required to refrain from strenuous exercise for four days prior to attending the laboratory. 

### 2.3. Equipment and Material

An isokinetic dynamometer (Cybex Norm, division of Lumex Inc., Ronkonkoma, New York, NY, USA) was used to passively rotate the knee joint where required (below). The angular position and velocity of the system input arm were produced at 100 Hz. For all trials, a portable B-mode ultrasound scanner (Echo Blaster 128 Ext-1Z system, Telemed, Vilnius, Lithuania) interfaced with a laptop via Echowave software (version II) (Telemed, Vilnius, Lithuania) was used to scan muscle fascicle/fibre length continuously at 40 Hz. The Reflotron Plus blood analysis machine (Roche Diagnostics GmbH, Mannheim, Germany) was used to analyse blood samples (below). Samples were taken using spring-loaded single-use 2 mm Unistik 3 lancets, Human GmbH 30 microlitre micropipettes, and Reflotron CK strips (Roche Diagnostics GmbH, Mannheim, Germany). The EMG system used to assess the pattern of muscle activity during the experiment was a Digitimer440 (Digitimer Ltd., Welwyn Garden City, Hertfordshire, UK), 4-channel system. A CED 1401 POWER3A (Cambridge Electronic Design Limited, Cambridge, UK) data acquisition system interfaced with Spike2 was used for data acquisition, allowing the synchronisation of EMG and US data collection. EMG signals were sampled at a frequency of 2000 Hz.

### 2.4. Experimental Protocol 

**Figure 1:**
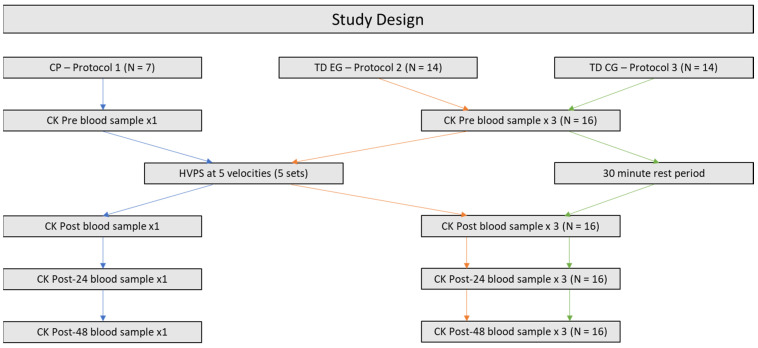
An outline of the experimental study design. Note. Blue arrows indicate Protocol 1, orange arrows indicate Protocol 2 and green arrows indicate Protocol 3.

The protocol of study is outlined in Figure 1 below. 

#### 2.4.1. Muscle-CK

CP participants (Protocol 1) underwent two blood samples that were taken using a standard finger-prick technique, and levels of muscle-CK were measured before (Pre) and immediately after (Post) the stretching protocol to provide some insight into the possibility of microscopic muscle fibre damage due to stretching. On the second (Post-24) and third (Post-48) days, 1 blood sample was taken from these participants while they were seated in a standard office chair which allowed monitoring of the level of the enzymes over 48 h from the imposed stretch. TD individuals in Protocols 2 and 3 underwent 6 blood samples on the first day, and levels of muscle-CK were measured before (Pre, 3 samples) and immediately after (Post, 3 samples) the stretching (TD-EG; Protocol 2) or rest period (TD-CG; Protocol 3). On the second (Post-24) and third days (Post-48), an additional 3 blood samples were taken from the participants in both TD groups (Protocols 2 and 3) while they were seated in a standard office chair (below). Such design allowed determination of typical error of measurement, estimation of minimal detectable change (MDC) and smallest clinically worthwhile change (SWC), and biological variation in muscle-CK over 48 h in the group. 

The Reflotron Plus blood analysis system was cleaned and checked according to manufacturing guidelines before use; a standardised warm-up period of two hours was undertaken. Blood samples of 30 microlitres were taken from the participants using a standard finger-prick technique; the finger was cleaned using a disposable alcohol wipe, and then pricked using a lancet. The blood sample was then collected using a micropipette and expelled onto a specialised CK strip, which was analysed by the Reflotron Plus blood analysis machine within 15 s of blood contact with the CK strip. This was performed pre and post passive stretching Protocol. As stated above, at Post-24 and Post-48, 1 sample was taken using the same standard finger prick technique for Protocol 1 (CP). A total of 3 samples were taken at each time-point for Protocols 2 and 3 (TD groups). A total of 4 blood samples (Protocol 1) and 12 blood samples (Protocols 2 and 3) were collected throughout 3 consecutive days. Results were expressed in microlitres per litre of blood. Muscular micro-damage was defined as microscopic damage to spastic and TD muscle fibres shown by the increase in CK concentration using two approaches: (1) more than 1.5 times the baseline concentration; and (2) more than 1.5 times the typical error above the upper limit of the confidence interval for the CK group mean. 

#### 2.4.2. Dynamometry 

**Figure 2:**
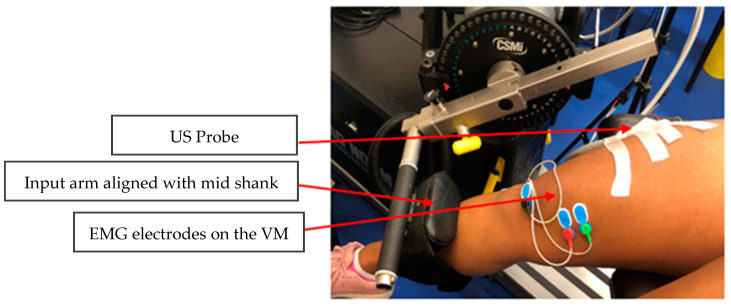
A participant’s leg with US and EMG, attached to the input arm of the dynamometer).

On the first day of attendance, CP participants were positioned on the isokinetic dynamometer lying supine, with the legs bent from the knee hanging from the edge of the chair. The most affected leg was attached to the input (moving) arm. The TD-EG participants were positioned similarly with the non-dominant leg attached to the dynamometry system input arm. The chair was adjusted for each participant so that the lateral epicondyle of the knee was aligned with the centre of rotation of the dynamometer arm. The resting knee angle was set at 90° flexion, and the hip angle was set at 0° of flexion (or as close as possible without causing discomfort). The cushion attached to the input arm of the dynamometer was aligned with the participants’ mid-shank and secured in place using Velcro straps (Figure 2). A gravity torque correction was performed during the dynamometry setup as part of the Cybex system setup. The range of motion (ROM) of the knee was adjusted for each participant: 0° was defined as the closest the participant could get to full extension of the knee. The continuous passive motion (CPM) of the dynamometer ranged from 0° to 90° of knee flexion, or the participants’ maximum ROM, whichever was less. CPM resulted in stretching of the quadriceps muscle during flexion of the knee joint; participants were instructed to relax during the stretching period. To prevent movement in the dynamometer chair, stabilisation straps were used across the trunk. 

Participants in Protocols 1 and 2 (CP and TD-EG) underwent a continuous passive stretching protocol at 5 different angular velocities. These were determined by the average and maximal angular velocities of the knee reported after gait analysis for the CP participants, but values were reported in the literature for TD-EG [27]. The chosen velocities for TD-EG increased in increments of 45°/s from the average angular velocity of the knee during walking (120°/s) to the maximum velocity of the dynamometer, 300°/s (i.e., 120°/s, 165°/s, 210°/s, 255°/s, 300°/s). Velocities used for the CP group increased in equal 20% increments from the average angular velocity of the knee during walking to the maximum angular velocity of the knee during walking, or the maximum velocity of the dynamometer (300°/s), whichever was less. A total of 2 practice (conditioning) rotations followed by three experimental rotations were performed at each velocity, with a 60 s rest period between each selected velocity. The order of the velocities of stretching was quasirandomised, beginning with the slowest velocity and ending with the fastest velocity for all participants, but was randomised in the middle. Ultrasound (US) images of the VL muscle were recorded during the experimental rotations for both groups. EMG activity of the vastus medialis (VM) and the biceps femoris (BF) muscles was recorded during the three experimental rotations at each angular velocity simultaneously only in Protocol 1 (CP). 

**Figure 3:**
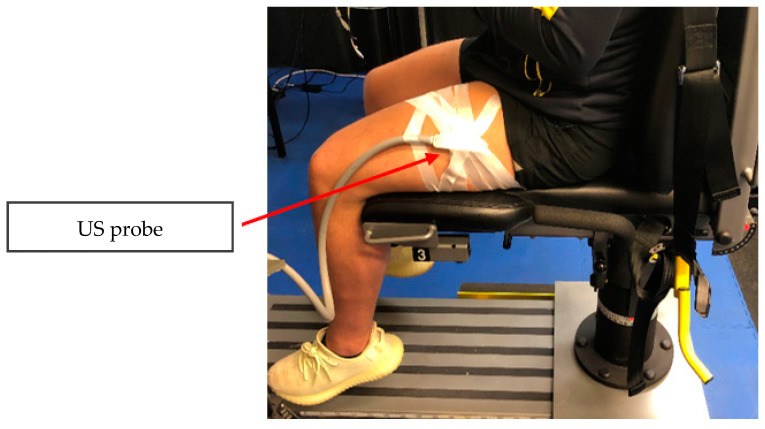
Image of a TD-CG participant with the probe attached during the rest period.

Participants in Protocol 3 (TD-CG) sat stationary in the dynamometer chair for 30 min (Figure 3) and did not receive any CPM of the knee joint. The chair was in a set position for all participants; the chair back was positioned upright so the participants’ hips were at 90°, and the participants’ non-dominant knee was hanging from the edge of the chair at approximately 90° and relaxed. Ultrasound (US) images of the VL muscle were recorded at the beginning and end of the rest period for approximately 3 s.

### 2.5. Electromyography Measures

EMG recorded in participants in Protocol 1 was used to monitor the presence of stretch reflex contractions of the VM muscle during movement. Electrode placement for the VM was eighty percent of the line between the ASIS and the knee joint space; the ground electrode was placed on the patella. 

### 2.6. Ultrasound Imaging

**Figure 4:**
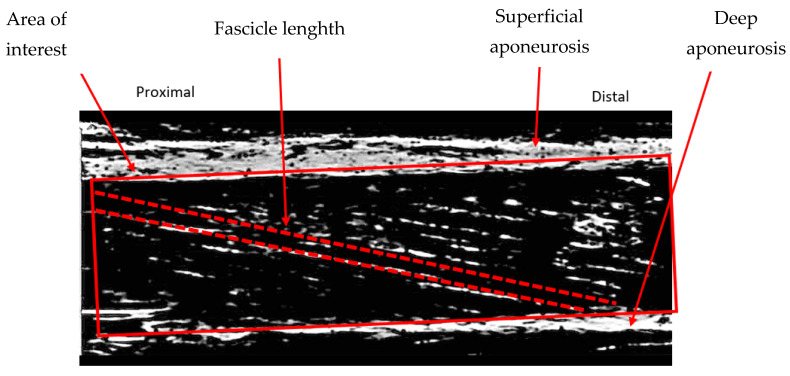
An ultrasound image of the VL of a CP participant.

For the TD individuals (Protocols 2 and 3), the US probe (5 cm scanning window) was attached midway between the greater trochanter and the femoral epicondyle, aligned with the line of action of the VL muscle fascicles and secured using hypoallergenic tape. For Protocol 1, different positioning of the US probe for each individual was required to obtain a clear reflection of the perimysial connective tissues representing fascicle orientation and length. Scanning depth and focus were optimised from person to person. VL fascicle length was defined as a straight line along the orientation of the muscle fascicles between the superficial and deep aponeuroses of the muscle within the area of interest (Figure 4). 

### 2.7. Data Processing and Analysis 

#### 2.7.1. Determining Fibre Strain

The US scan was started prior to the beginning of the three experimental rotations. The time lag between the start of US scanning and synchronous recording of the EMG and dynamometry data was between 70 ms and 90 ms (scan rate was 40 frames per second). US data were collected in .tvd format and saved and exported in .avi format, then opened in MATLAB (R2018b, MathWorks, Natick, MA, USA). The length changes of a defined muscle fibre were tracked. Fibre strain was calculated for the flexion phase (Protocols 1 and 2) of the knee rotation. Associated changes in the VL fascicles, measured with respect to their reference length (FL origin), were measured using a custom-written algorithm in MATLAB (R2018b, MathWorks, USA) and used in the calculation of strain (Equation (1)).

Strain = (max FL − FL origin)/FL origin(1)

where FL origin was defined as the length of the muscle fascicle/fibre at full extension of the knee when no extension torque was recorded, and max FL was the maximum length of the fascicle during flexion of the knee joint. 

Each rotation of the knee joint was identified using dynamometry system maximum and minimum position (angle) traces, and then separated into flexion and extension phases. 

#### 2.7.2. Determining Changes in Muscle Activation

EMG was recorded to establish whether (a) reflex contraction would occur in the stretching quadriceps during knee flexion (particularly in individuals with CP); and (b) whether such reflex contraction would subsequently subside (drop to the baseline muscle activity or below it). EMG data were demeaned to remove DC offset, rectified, and smoothed based on a median value of each waveform in Spike2 before being exported as a text (.txt) file and analysed using a custom-written algorithm in MATLAB. 

Baseline muscle activity was defined as the Average Rectified Value (ARV) calculated over the duration of knee extension. The first and last 100 ms of the dynamometry extension period were not included as part of the extension period for the calculation of ARV as it was associated with the changing direction of the dynamometry input arm from flexion to extension, or vice versa, at the end of flexion and extension phases, respectively. Muscle activity during this time could have also been affected by the expectation of the movement coming to a halt prior to the change in direction. 

ARV during knee flexion was calculated and examined using successive (non-overlapping) epochs of 60 ms for the duration of the flexion phase [23]. The reflex contraction was identified when the ARV for an epoch of 60 ms was larger than the baseline ARV by 25% (i.e., >baseline + 25%baseline). Muscle was considered as “inhibited” if any increased ARV activity dropped back to the baseline in any epoch of 60 ms. Muscle was considered as “deactivated” if the ARV dropped below the baseline by 25% (i.e., <baseline − 25% baseline) in any epoch of 60 ms. We expected that TD-EG would not show any increased quadriceps activity during knee flexion at any velocity of knee rotation hence we did not record EMG in this group [28], while reflex contraction would be observed during knee flexion in the spastic muscles. We did not make any prediction if the reflex contraction would be maintained or inhibited/deactivated throughout the flexion phase in this group. However, if the former situation occurred, we would mimic eccentric exercise, while if the latter situation occurred, it would be similar to the clasp-knife phenomenon in CP. 

#### 2.7.3. Determining Changes in Muscle-CK

Changes in the level of blood muscle-CK, identified by our minimally invasive technique of finger-pricking, were used to establish whether biological variation in this parameter occurred, whether any microdamage due to intervention occurred, and whether there were any differences between individuals with and without CP in response to stretching:

- Determination of biological variation in muscle-CK: Standard deviation of changes in the levels of muscle-CK (change score) at different time points (i.e., between Pre and Post stretching, Pre and Post-24, and Pre and Post-48) was used in the calculation of standard error of measurements (SEM) for each group (Equation (2)).

The highest Minimal Detectible Change (MDC_95_) that could be calculated based on the SEM across all comparisons made from individuals in Protocol 3 (TD-CG) would be the most conservative value to determine changes in the blood muscle-CK due to biological variations and device error (Equation (2)). 

An estimate of the smallest worthwhile change (SWC) in muscle-CK was also calculated (Equation (2)) based on values obtained from TD-CG participants.

Moreover, in the absence of any better alternative, the MDC_95_ and/or SWC from individuals in TD-CG could provide a ballpark figure for assessing alterations in the resting state of the enzyme in individuals with CP in response to any interventions that may microscopically damage the spastic muscles.

SEM = SD/√2
MDC_95_ = SEM × √2 × 1.96
LoA = ∆score ± MDC
SWC = SD × 0.2(2)

where SEM is the standard error of measurement, SD is the standard deviation of the change scores (∆score − change in the mean value of the measured muscle-CK at different time points), MDC_95_ is the (highest value) minimum detectable change calculated using SEM obtained across all possible comparisons in Protocol 3, LoA is the level of agreement (confidence interval), and SWC is the smallest worthwhile change.

- Determination of muscle microdamage and differences between individuals with CP and healthy individuals in response to stretching: All analyses were conducted in SPSS (IBM SPSS Statistics 25.0). A between-subjects ANOVA with repeated measures was conducted on the change in muscle-CK (change score) at 3 time points (i.e., between post, 24 h and 48 h and baseline values) of Protocols 2 and 3 in order to examine the effect of stretching in the healthy and CP individuals over time. Baseline (pre) values of the muscle-CK were used as covariates. To this end, we obtained one measure of blood muscle-CK from individuals in Protocol 1 (CP), and 3 from those in Protocol 2 (TD-EG). For this group, the mean of the 3 measurements at each time point was used in the analysis.

We had decided to use the MDC_95_ and/or the SWC from Protocol 3 (TD-CG) to establish if any observed difference in the change score of muscle-CK at different time points in Protocols 2 and 3 could be attributed to stretching and not biological variation or device error. 

#### 2.7.4. Measures of Reliability

For muscle-CK, 3 measures of reliability, change in mean (change score), typical error, and coefficient of variation were calculated for healthy individuals in Protocol 2 (*n* = 9) and Protocol 3 (*n* = 7) at Post, Post-24, and Post-48 compared to baseline (Pre) values. Intraclass Correlation Coefficients (ICC) were calculated following Hopkins, 2015 [29] to measure the degree of consistency of the blood muscle-CK values at each time point. The ICC was also calculated for the fascicle lengths to assess the experimenter’s reliability when measuring maximum and minimum fascicle lengths during the three random experimental rotations of the knee joint for each participant in TD-EG participants. 

## 3. Results

Mean values for muscle-CK at each time point for individuals in Protocols 1–3 are shown in Table 1. A total of 1 participant in Protocol 3 had extremely high muscle-CK values (>1000 μL/L) and the blood analyser produced an error message (dilute sample). Data of this participant were removed.

Changes in the mean values from baseline (Δscore) for individuals in Protocols 1–3 can be seen in Table 2. 

Measures of variability for muscle-CK, estimated based on the values obtained from healthy individuals in Protocol 3 (TD-CG), are shown in Table 3. MDC_95_ and SWC values increased at each time point. The highest value for MDC_95_ (273.3 μL/L) and associated LoA (−234.2–312.4 μL/L) provided the most conservative estimation of biological variation in muscle-CK. These values should be treated with caution as they were mainly driven by an extremely high value obtained from Participant 16 (in bold in Table 2). With the exclusion of the change scores at the last 2 time points of participant 16, MDC_95_ and LoA would change to 71.2 μL/L; and [−69.9–72.5] at 24 h, and 50.4 μL/L; [−76.4–24.4] at 48 h (Table 3). 

Measures of muscle-CK variability for individuals in Protocols 1 and 2 are in Table 4. As a group, individuals in Protocol 2 (TD-EG) showed an overall small drop immediately post-intervention but a small increase in muscle-CK levels at Post-24 and Post-48 time points. As a group, individuals with CP showed an overall small increase in muscle-CK levels immediately post-intervention. At Post-24 and Post-48 time points, however, muscle-CK values were smaller than the baseline values. Not all individuals in Protocols 1 and 2 followed a similar pattern of change to their respective group (Table 1). 

Immediately after stretching, measured muscle-CK increased in two participants in the CP group, and five participants in the Healthy group. Others showed a drop in the measured muscle CK. After 24 h, 2 participants in the CP group and 2 healthy participants had positive muscle-CK change score values. After 48 h, 3 CP participants and 4 healthy participants had positive muscle-CK change score values. In neither situation, the increase in muscle-CK after stretching was above the upper bound of the LoA (312.4 or 24.4 μL/L) established based on healthy participants in Protocol 3 (TD-CG).

An independent samples *t*-test showed no significant differences in the muscle-CK values at the baseline between individuals in Protocols 1 and 2 (t_(13)_ = −0.020; *p* = 0.985; *CI* [−74.79–73.43]. Moreover, there was no difference in their muscle-CK response to stretching (Table 5). For this latter analysis, muscle-CK change scores at different time points with respect to baseline were used in the ANOVA with baseline measures from participants entered into the model as covariates. Group (Protocol 1 vs. Protocol 2) was the between-groups factor. The results of the analysis are shown in Table 5.

### 3.1. Fibre Strain

Table 6 shows the maximum strain observed during stretching of the MTU and the associated speed at which the maximum strain occurred for participants in Protocols 1 and 2. All participants in both groups experienced positive fibre strain in response to passive stretching at different velocities; however, an increase of 5% (a subjectively decided minimum value to show non-trivial positive strain) or more in the length of fascicles at any time during stretching (i.e., flexion of the knee joint) with respect to its initial (origin) length (i.e., when the knee joint was at its maximum extension angle) was observed in a limited number of participants. As illustrated in the table below, 4 participants in Protocol 1 (CP) experienced a maximum positive fibre strain of 5% or more during stretching. Maximum fibre strain ranged between 0.67% and 45.27% in this group. Positive fibre strain of ≥5% was observed in 4 healthy participants (Table 6). The majority of maximum strain was recorded at relatively lower velocities (speeds 1–3; i.e., up to 75% above the average knee angular velocity recorded during walking at self-selected speed in CP, and up to 210 °/s in healthy individuals). As a group, individuals with spastic muscles experienced greater average maximum strain than typically developing individuals (11% vs. 5%); however, there were large within-group variations in these responses to stretching protocol, and the higher number for the CP group was driven by 1 participant (participant 3; 45%). For this participant, the maximum strain in other trials was 4%. 

An intraclass correlation coefficient was used to assess the experimenter’s reliability when measuring maximum fascicle length during three rotations at the slowest and three rotations at the fastest velocities recorded for participants in Protocol 2. Maximum FL measurements at both slow and fast velocities showed strong to moderate correlations when comparing rotations, ranging from 0.96 to 0.65, respectively (Table 7).

### 3.2. VM Muscle Activity during Stretching

**Figure 5:**
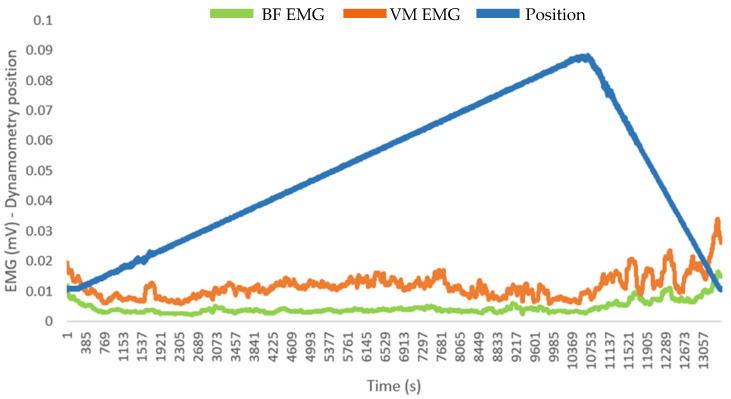
The EMG of the VM and BF plotted superimposed onto the position of the shank represented by dynamometry data. Note. Position trace has been rescaled to fit with the EMG activation signal. Units of time are arbitrary.

An example of the EMG output from a single rotation in a participant with CP can be seen in Figure 5. Positive and longer slope of the position trace represents the extension of the knee joint from its most flexed position to full extension, which occurred at a constant velocity of 15 degrees/second. The negative and shorter slope of the position trace represents flexion of the knee. 

VM muscle activity during knee extension was expected to be minimal in the TD-EG participants as the VM was shortening during knee extension, assuming that the participant could follow the instructions and remain relaxed. For the participants with CP, we expected reflex contraction, particularly at higher velocity(s) of joint flexion (lengthening of the MTU), which could be interspersed with periods of decreased activity (deactivation or inhibition) due to the clasp-knife phenomenon. 

Out of the three experimental rotations, the final two were analysed for the results section. The state of VM muscle activity during stretching, i.e., during flexion of the knee joint, for participants in the CP group at all speeds is illustrated in Table 8. VM reflex activation for at least 1 epoch of 60 ms did not occur at 2 speeds (Speed 1 and Speed 3) in 1 participant (P7), and at the slowest velocity of rotation (Speed 1) in 2 other participants (P12 and P13). All participants experienced at least 1 epoch (60 ms) of increased activation of the VM during knee flexion at higher velocities (Speed 4 and/or Speed 5) in at least one of the experimental rotations. 

All participants but one (P9) in whom reflex contraction was maintained throughout joint flexion at the highest velocity (Speed 5) experienced at least one epoch of inhibition and/or deactivation. The inhibition/deactivation occurred between 180 ms and 360 ms into the flexion period. EMG was not measured in Protocol 2 as healthy individuals were not expected to experience a reflex contraction during passive stretching. 

## 4. Discussion

The purpose of this exploratory, non-confirmatory study [30] was to examine whether the three presumed criteria for increasing muscle fascicle length in healthy muscles [19], associated with high-velocity eccentric training, could be met by undergoing passive stretching of the muscles at (appropriately) high velocities. We compared the behaviour of spastic and healthy quadriceps during passive flexion of the knee joint under different isokinetic velocities and examined the strain of the fascicles, blood markers of muscle damage, and muscle electrical activity. We established the minimal detectable change and/or smallest worthwhile change of blood CK levels as a result of stretching, not confounded by biological variation or device error, in a group of healthy participants to determine, via generalisation to the CP group, whether the passive stretching protocol in spastic muscles resulted in sufficient muscle damage. The experimenter reliability in measuring TD muscle fascicle length using ultrasonography was also determined.

We assumed passive stretching of the spastic muscle-tendon unit (MTU) at an appropriately high velocity would induce (stretch) reflex contraction, and hence, with continuation of stretching throughout the available knee range of motion, resemble eccentric exercise in healthy individuals and may lead to muscle damage. 

Positive strain of the MTU fascicles is an important factor in predicting muscle fibre damage with stretching. Greater mechanical strain associated with high muscular contraction force, similar to the condition of exercise under an eccentric regime, likely results in greater damage to contractile proteins [7,31]. Stretching of the MTU is commonly practiced within CP to maintain or improve the available range of motion with the added assumption that maintaining stretch around the extreme ROM of the joint may result in an increase in muscle fascicles in the long term [4,5,32,33]. All participants with CP who underwent stretching experienced positive fascicle strain to varying degrees (1–45%) (Table 6), suggesting the current experimental protocol could at least provide the minimal requirement of regular stretching interventions for increasing ROM and FL with no adverse effect. 

For obtaining higher strain in the MTU during flexion of the joint and creating a larger possibility of inducing microscopic damage to the muscle during exercise, fascicle length was measured with the participants lying supine with the knee joint of the plinth of the dynamometry system, but with the ROM limited to the maximum available or 90 degrees, whichever was less. Under this experimental condition, many of the muscles in both groups were either contracting slightly in expectation of the upcoming flexion movement or experiencing continued reflex contraction from the previous stretch (pre-loading). Therefore, strain values, rather than reflecting the maximum strain possible in individual muscles, reflected the maximum determined using alteration in the length of fascicles within the viewing probe under the experimental condition of the present study. 

Our findings suggest that a single session of passive stretching, following the protocol employed in the current study, can induce positive fascicle strain in spastic muscles; however, there could be other than methodological factors that dictate the amount of strain that muscles/fascicles experience. The level of spasticity or length and relative compliance of the muscle compared to the tendon being stretched might have contributed to the observed variation in values in CP [8]. It has previously been reported that compliance of the spastic tendon may be higher than its corresponding muscle [34,35], which may mean the relative stretch experienced by the spastic tendon could be higher than the spastic muscle/fascicles during exercise, and lead to less fibre strain and subsequent damage. Positive fibre strain during stretching is assumed to be required for damage to a muscle and trigger sarcomerogenesis [19,36]. Observed alterations in FL during exercise satisfy the first of three presumed criteria for increasing FL with training in this population [19].

The literature on the matter of biological variability of muscle-CK is inconsistent: demographic factors such as gender, age, ethnicity, as well as activity level all cause baseline CK variation, causing inconsistency regarding acceptable levels of CK as a result of exercise [37]. The minimal detectable change and smallest worthwhile change for blood CK have previously been reported as 1.5 times baseline levels, or 3 times baseline levels in extreme cases, such as myocardial infarction [38]. However, the minimal detectable change increased at each time point, meaning as time progresses, a higher rate of increase in CK is necessary to indicate muscle damage occurs due to intervention and not increases in biological variation. The literature reporting CK variation over time as a result of resistance training intervention shows increases in CK peak 24–72 h after intervention occurs. Our results, however, do not reflect this (Table 1, Table 2, Table 3 and Table 4). 

We decided to use the most conservative value of the increase in muscle-CK calculated in TD-CG individuals (312.4 μL/L or 24.4 μL/L; Table 3) to inform protocols used in CP populations where determination of the level of muscle damage due to passive stretching is an outcome measure. Patterns of alteration in muscle-CK post-stretching in CP and TD-EG participants were inconsistent. Some participants showed no or minimal increase as a result of stretching, which could be interpreted as the ineffectiveness of Protocols 1 and 2 in inducing muscle micro-damage in typically developing or spastic muscles. In a few other participants, values of muscle-CK dropped after 24 or 48 h. 

Moreover, even when the smaller of the two conservative values for the upper bound of LoA was used, an increase in muscle-CK was seen only in two participants in the CP and TD-EG groups. No difference in the muscle-CK levels was found between individuals in Protocols 1 and 2 at baseline, or in their response to intervention either (Table 5). Bias due to missing data or selection of participants in the study might have also affected muscle-CK outcomes.

Collectively, the second criterion for increasing FL during exercise—i.e., damage to muscle quantified by measuring muscle-CK using finger-pricking—was not met under the current experimental protocol, and due to the number of missing values and bias which may have been introduced due to dropping one participant at the outset from Protocol 2, we interpret these results as being inconclusive.

Stretching of the spastic VM at appropriately high velocity resulted in a reflex contraction in participants with CP, which was followed by at least one epoch of inhibition or deactivation. As expected, individuals with CP experienced the occurrence of reflex contractions during stretching at the highest speeds. Such contraction is assumed to be a protective mechanism that occurs in spastic muscles to prevent muscle damage occurring due to overstretching [11,39]. The reflex contractions during passive stretching protocol throughout ROM imposed by the dynamometry system indicated that muscle was resisting the stretch, and, therefore, passive stretching exercise protocol in CP could be considered similar to eccentric exercise in healthy participants. While eccentric training in spastic muscles may not be possible due to the inability of the participants to selectively control the stretching muscle during exercise, the present study provides an experimental paradigm for eccentric training within the CP population with no requirement for their active participation. This could be hugely beneficial to the population, as eccentric training may increase muscle fascicle length, and subsequently alter force-length and force–velocity characteristics of the MTU and lead to improved effectiveness and efficiency of muscle capacity for producing force during functional activities. 

Reflex contraction which occurred during passive stretching at the relatively higher velocity (Velocities 4 and 5) was followed by a drop in the activation of muscle in all but two participants (Table 8). A drop in the reflex muscle activation could be attributed to the clasp-knife phenomena, in which the muscle gives way to the stretch to prevent further damage to the muscle. This could indicate that the individuals who experienced reflex contraction followed by a drop in the activation could benefit from repeated sessions of passive stretching training, as the muscle would be sufficiently damaged with repeated episodes of eccentric-like stretch. Such training could theoretically lead to sarcomerogenesis and subsequently increase muscle FL and induce functional adaptations in these individuals. Further investigation is required to establish if our proposed experimental paradigm, i.e., mimicking eccentric training in the TD population via long-term high-velocity passive stretching could actually lead to increased muscle FL and result in functional adaptations in individuals with CP. Moreover, it seems the current experimental protocol can satisfy the third of the suggested criteria, i.e., a momentary drop in the level of activity of stretching muscle for increasing FL with training in CP [19]. 

ICC values, to assess experimenter reliability in measuring VL FL, showed strong correlations at different velocities, supporting that the experimenter was reliable at measuring TD muscle FL. Reliably measuring FL in CP is hugely important as increasing FL is an important outcome measure of interventions for the population. Due to the uniqueness of muscle shape in every participant with CP, reliability of measurement of FL (and/or other architectural parameters of interest) must be established in a number of baseline measurements prior to starting intervention to alter FL, and not confounded by the errors of measurement. In the present study, participants with CP only attended a one-off stretching session, and hence, establishing day-to-day measurement reliability was not required. 

### Limitations

A sample of convenience from participants who belonged to a wide range of ages and functional abilities was used. Measures of muscle-CK reported by the analyser were erroneous (a constant number was reported) for some blood samples and led to missing data. We identified that the likely underlying reason was an insufficient spell of blood on the CK strip. No consistent increases in CK were observed, implying that the stimulus may not have been sufficient in the TD population. We did not deviate from the intended intervention during the study in either protocol and no participant, apart from P7 at Speed 5, showed any adverse effects to the intervention employed. Researchers were not blinded to the outcome measures but employed the same method for the analysis of data across participants/groups. 

## 5. Conclusions

It is likely from the data presented here that at least two of the three presumed criteria necessary to increase healthy muscle fibre length can be met using a single bout of high-velocity passive stretching in individuals with CP. Whether muscle micro-damage could occur as a result of exercise was, however, inconclusive. Future interventions in individuals with CP could use passive stretching at high velocities as a replacement for eccentric training, with the view to increase muscle fibre length and improve function over time. 

## Figures and Tables

**Table 1:** Mean muscle-CK values at different time points for individuals in Protocols 1–3.

Group	P No.	Pre	Post	24 h	48 h	Notes
Protocol 3—C	3	42.6	46.4	45.3	42.7	
Protocol 3—C	4	87.9	105.9	86.1	74.5	
Protocol 3—C	6	136	81.2	188.3	M	Missed appointment
Protocol 3—C	9	70.7	122.7	138.7	114.3	
Protocol 3—C	11	128.7	108.3	98.5	104.7	
Protocol 3—C	12	240.3	220	M	M	Felt sick
Protocol 3—C	16	64.8	51.4	125.3	410	
Group Mean		110.1	105.1	113.7	149.2	
Protocol 2—E	1	59.5	71	59.1	59	
Protocol 2—E	2	118.7	147	110.5	90.6	
Protocol 2—E	7	53.3	60.7	63.3	47.1	
Protocol 2—E	8	63.6	51.8	61.8	67.7	
Protocol 2—E	10	156.7	152.3	327.7	286.3	
Protocol 2—E	13	190.7	207.7	182	147.3	
Protocol 2—E	14	97.1	133.3	79.9	107.8	
Protocol 2—E	15	155	137.3	125.3	104.1	
Protocol 2—E	17	246.7	166.2	239.3	333.7	
Group Mean		126.8	125.3	138.8	138.2	
Protocol 1—CP	3	191	179	144	78.2	
Protocol 1—CP	4	39.5	M	71.7	51.6	
Protocol 1—CP	6	56.3	M	48	58.1	
Protocol 1—CP	7	173	199	217	192	
Protocol 1—CP	9	160	121	M	145	
Protocol 1—CP	12	137	195	114	88.1	
Group Mean		126.1	173.5	118.9	102.2	

Note. Group = protocol number, control (TD-CG), exercise (TD-EG), or cerebral palsy (CP), P No. = participant number, pre = mean CK values pre-stretching, post = mean CK values post-stretching, 24 h = mean CK values 24 h post-stretching, 48 h = mean CK values 48. Muscle-CK values are in μL/L, M = missing datum.

**Table 2:** Changes in muscle-CK mean values at each time-point for individuals in Protocols 1–3.

Group	P No.	Δscore Post-Pre	Δscore 24 h-Pre	Δscore 48 h-Pre
Protocol 3—C	3	3.8	2.7	0.1
Protocol 3—C	4	18	−1.8	−13.4
Protocol 3—C	6	−54.8	52.3	M
Protocol 3—C	9	52	68	43.6
Protocol 3—C	11	−20.4	−30.2	−24
Protocol 3—C	12	−20.3	M	M
Protocol 3—C	16	−13.4	**60.5**	**345.2**
Protocol 2—E	1	11.5	−0.4	−0.5
Protocol 2—E	2	28.3	−8.2	−28.1
Protocol 2—E	7	7.4	10	−6.2
Protocol 2—E	8	−11.8	−1.8	4.1
Protocol 2—E	10	−4.4	171	129.6
Protocol 2—E	13	17	−8.7	−43.4
Protocol 2—E	14	36.2	−17.2	10.7
Protocol 2—E	15	−17.7	−29.7	−50.9
Protocol 2—E	17	−80.5	−7.4	87
Protocol 1—CP	3	−12	−47	−112.8
Protocol 1—CP	4	M	32.2	12.1
Protocol 1—CP	6	M	−8.3	1.8
Protocol 1—CP	7	26	44	19
Protocol 1—CP	9	−39	−160	−15
Protocol 1—CP	12	58	−23	−48.9

Note. Group = protocol number, control (TD-CG), exercise (TD-EG), or cerebral palsy (CP), P No. = participant number, Δscore post-pre = change in the mean of the values from Pre to Post time point, Δscore 24 h-pre = change in the mean of the values from Pre to 24 h time point, Δscore 48 h-Pre = change in the mean of the values from Pre to 48 h time point. Muscle-CK values are in μL/L, M = missing datum. Values in bold indicate potential anomalies.

**Table 3:** Measures of variability in muscle-CK for TD-CG individuals in Protocol 3.

Measure	Post-Pre	24-Pre	48-Pre	
∆ in Mean	−5	3.6	39.1	
SD	31.3	36.8	139.4	
SEM	22.1	26	98.6	
CV	10.0%	10.0%	30.0%	
MDC_95_	61.3	72.1	273.3	
SWC	25.07	32.78	136.58	
LoA	[−66.3–56.3]	[−68.5–75.7]	[−234.2–312.4]	
		**[−69.9–72.5]**	**[−76.4–24.4]**	

Note. Post-Pre = change in the mean of the values measured at Post and Pre time points, 24-Pre = change in the mean of the values measured at Post-24 and Pre time points, 48-Pre = change in the mean of the values measured at Post-48 and Pre time points, ∆ in Mean = change in mean of repetitions at each time point, SD = standard deviation of Δ in Mean, SEM = Standard Error of Measurement, CoV = Coefficient of Variation, MDC = Minimal Detectable Change, SWC = Smallest Worthwhile Change, LoA = Level of Agreement. Please see text for highlighted LoA values. Values in bold are after the removal of P16.

**Table 4:** Measures of muscle-CK variability for typically developing individuals in Protocols 1 and 2.

Measure	Group	Post-Pre	24-Pre	48-Pre
∆ in Mean	Protocol 2	−1.5	12	11.4
SD	Protocol 2	32.5	57.2	56.4
SEM	Protocol 2	23	40.4	39.9
CV	Protocol 2	30.0%	50.0%	40.0%
∆ in Mean	Protocol 1	8.3	−8.7	−39.4
SD	Protocol 1	36.9	38.5	48.7
SEM	Protocol 1	26.1	27.2	34.4
CV	Protocol 1	20.00%	20.00%	30.00%

Note. ∆ in mean = change in mean at each time point, SD = standard deviation of Δ in Mean, SEM = Standard Error of Measurement, CV = Coefficient of Variation, MDC = Minimal Detectable Change, SWC = Smallest Worthwhile Change, LoA = Level of Agreement. Post-Pre = mean change of the values measured at Post, 24-Pre = mean change of the values measured at Post-24, 48-Pre = mean change of the values measured at Post-48.

**Table 5:** Results of multivariate ANOVA on change scores (Δscore) between CP and TD-EG groups at different time points (post, after 24, and 48 h).

Statistic	df	Mean Square	F	Sig.	η_p_^2^
Time Point	2	4510.901	1.977	0.165	0.165
Time Point × Baseline	2	3428.977	1.503	0.247	0.131
Time Point × Group	2	5886.618	2.580	0.101	0.205

Note. Time point = post, 24 h, and 48 h, Timepoint × Baseline = time point interaction with Baseline, Timepoint × Group = timepoint interaction with TD or CP group.

**Table 6:** Vastus lateralis maximum fibre strain.

Vastus Lateralis FL Strain in CP			Vastus Lateralis FL Strain in TD-EG		
Participant	Speed	Max Strain	Participant	Speed	Max Strain
3	5	45%	1	4	1%
4	1	2%	2	1	8%
6	3	6%	7	1	1%
7	3	9%	8	3	17%
9	3	8%	10	1	3%
12	1	4%	13	3	6%
13	5	1%	14	3	3%
			15	2	2%
			17	5	1%
			20	2	1%
			24	1	3%
			27	2	19%
			28	1	1%

Note: Speed = velocity at which the largest fascicle strain occurred is reported, with 1 being the slowest and 5 being the fastest velocities used during knee flexion. Max Strain = maximum FL strain observed during knee flexion expressed as a percentage of VL origin length.

**Table 7:** Intraclass correlation coefficient measuring experimenter reliability.

Intraclass Correlation Coefficient Measuring Experimenter Reliability					
Trials	2-1	3-2	4-3	5-4	6-5
Intraclass correlation (ICC)	0.95	0.94	0.65	0.95	0.94
Lower conf. limit	0.82	0.83	0.18	0.83	0.83
Upper conf. limit	0.98	0.98	0.88	0.98	0.98

Note. Trials = which of the rotations is being compared (1–3 being slowest, 4–6 being fastest), ICC = correlation score for the trials being compared.

**Table 8:** State of VM muscle EMG activity during knee flexion at different velocities in Protocol 1 (CP participants).

	Speed 1	Speed 2	Speed 3	Speed 4	Speed 5	Candidate
P3	Y:Y	Y:Y	Y:Y	Y:Y	Y:Y	C
	Y:N	Y:Y	Y:Y	Y:Y	Y:Y	
P4	Y:Y	Y:Y	N:N	Y:Y	Y:Y	C
	Y:Y	Y:Y	Y:Y	N:N	Y:Y	
P6	Y:Y	Y:Y	Y:Y	Y:Y	Y:Y	C
	Y:Y	Y:Y	Y:Y	Y:Y	Y:Y	
P7	N:N	Y:Y	N:N	N:N	Missing	UNDECIDED
	N:N	N:N	N:N	Y:Y	Missing	
P9	Y:Y	Y:Y	Y:Y	Y:Y	Y:N	C
	Y:Y	Y:Y	Y:Y	Y:Y	Y:N	
P12	N:N	N:Y	N:Y	N:Y	Y:N	C
	N:N	Y:N	N:Y	N:N	N:Y	
P13	N:Y	N:N	N:N	N:N	Y:N	C
	N:N	Y:N	Y:N	Y:N	Y:N	

Note: The first letter (Y or N) in a pair shows whether or not flexion of the knee joint resulted in increased muscle activation (reflex contraction) for at least 60 ms anytime during joint flexion (i.e., increased ARV above the threshold for at least one epoch of 60 ms). The second letter (Y or N) shows whether or not any such increased activity was followed by at least 60 ms of inhibition/deactivation. The two rows of results for each participant belong to the last two experimental rotations used in the analysis. Based on the state of muscle activity during stretch, we decided whether a participant was a candidate for training (C) (i.e., whether they experienced a reflex contraction followed by a drop in activation during the highest velocity). Participant 7 did not tolerate speed 5, and, therefore, data are missing for this condition.

## Data Availability

Data is available upon request from the corresponding author.
